# INFLUENCE OF HEPATOCELLULAR CARCINOMA ETIOLOGY IN THE SURVIVAL AFTER RESECTION

**DOI:** 10.1590/0102-6720201600020010

**Published:** 2016

**Authors:** Felipe de Lucena Moreira LOPES, Fabricio Ferreira COELHO, Jaime Arthur Pirolla KRUGER, Gilton Marques FONSECA, Raphael Leonardo Cunha de ARAUJO, Vagner Birk JEISMANN, Paulo HERMAN

**Affiliations:** Liver Unit, Department of Gastroenterology, University of São Paulo Medical School, São Paulo, SP, Brazil

**Keywords:** Carcinoma, hepatocellular, Hepatectomy, Survival analysis, Etiology, Prognosis

## Abstract

**Background:**

Hepatocellular carcinoma (HCC) is the most frequent type of primary liver cancer and its incidence is increasing around the world in the last decades, making it the third cause of death by cancer in the world. Hepatic resection is one of the most effective treatments for HCC with five-year survival rates from 50-70%, especially for patients with a single nodule and preserved liver function. Some studies have shown a worse prognosis for HCC patients whose etiology is viral. That brings us to the question about the existence of a difference between the various causes of HCC and its prognosis.

**Aim:**

To compare the prognosis (overall and disease-free survival at five years) of patients undergoing hepatectomy for the treatment of HCC with respect to various causes of liver disease.

**Method:**

Was performed a review of medical records of patients undergoing hepatectomy between 2000 and 2014 for the treatment of HCC. They were divided into groups according to the cause of liver disease, followed by overall and disease-free survival analysis for comparison.

**Results:**

There was no statistically significant difference in the outcomes of the groups of patients divided according to the etiology of HCC. Overall and disease-free survival at five years of the patients in this sample were 49.9% and 40.7%, respectively.

**Conclusion:**

From the data of this sample, was verified that there was no prognostic differences among the groups of HCC patients of the various etiologies.

## RESUMO

**Racional:**

O carcinoma hepatocelular (CHC) é o mais frequente tipo de câncer primário do fígado e a sua incidência vem aumentando nas últimas décadas, tornando-o hoje a terceira causa de morte por câncer no mundo. A ressecção hepática é um dos tratamentos mais eficazes para ele com taxas de sobrevida em cinco anos de 50-70%, especialmente para pacientes com nódulo único e função hepática preservada. Alguns estudos mostraram pior prognóstico para os pacientes com CHC cuja causa é a infecção por vírus B ou C. Isso leva à questão sobre a existência de possível diferença entre as diversas causas e o prognóstico.

**Objetivo:**

Comparar o prognóstico (sobrevida global e livre de doença em cinco anos) de pacientes submetidos à hepatectomia para o tratamento do CHC com relação às diversas causas da hepatopatia.

**Método:**

Foi realizado levantamento de prontuários dos pacientes submetidos à hepatectomia entre 2000 e 2014 para tratamento de CHC. Eles foram divididos em grupos de acordo com a causa da hepatopatia, sendo feita análise de sobrevida para comparação.

**Resultados:**

Não houve diferença estatisticamente significante de prognóstico entre os grupos de pacientes divididos conforme a causa do CHC. A sobrevida global e livre de doença em cinco anos foi de 49.9% e 40.7%, respectivamente.

**Conclusão:**

Pôde-se constatar que não houve diferença em relação ao prognóstico entre os grupos de pacientes das diversas causas de CHC.

## INTRODUCTION

Hepatocellular carcinoma (HCC) is the most common type of primary liver cancer and its incidence has increased worldwide in recent decades, making it currently the fifth most common form of malignancy in men and the ninth in women with a men/women ratio of 2.4^5^.

Every year there are approximately 700,000 to 1,000,000 new cases and about 600,000 to 800,000 of them die from the disease, making HCC the second leading cause of death by cancer in the world^10^.

Resection remains one of the most effective treatments with five-year survival rates from 50-70%, especially for patients with a single nodule and preserved liver function^6^.

The development of science is leading us to realize that diseases and therapies must be individualized. Huge differences can be observed on the biological behavior of the same disease, very aggressive in ones and insidious in others^2^ ^,^ ^6^ ^,^ ^17^. In this context, the study of prognostic factors is important to identify patients with an aggressive disease and, consequently, adapt the therapy accordingly.

A few studies have demonstrated a poor prognosis for patients whose etiology of HCC was virus B or C infection. Due to the negative impact on prognosis, some even suggest primary liver transplantation for those patient^2^ ^,^ ^4^ ^,^ ^12^ ^,^ ^20^.

Due to this difference in prognosis between viral and non-viral etiology, is proposed a study to evaluate the prognosis of patients submitted to HCC resection, according to the etiology of the disease.

## METHOD

Was gathered data from patients with HCC single nodule, who underwent resection, between August 2000 and July 2014. This study was approved by the University Ethics Committee.

The analysis included only patients with pathological confirmation of HCC who underwent hepatectomy with curative intent. Patients with fibrolamellar HCC and hepatocholangiocarcinoma were excluded. Thus, 101 patients were the subjects of this study. 

Patients were divided into groups according to the etiology of the liver disease: HCV (n=34), HBV (n=11), alcohol (n=13), NASH (n=8), mixed etiology (n=14), representing HBV, HCV and alcohol, with at least two of those three; and other etiologies (n=21), including cryptogenic, hemochromatosis, autoimmune hepatitis and others.

For all groups, an analysis of overall and disease-free survival at five years was performed, to establish whether the cause of liver disease influenced the prognosis.

The Kaplan-Meier method was used to evaluate the overall and disease-free survival, followed by the Log-Rank test to compare the curves.

For all conclusions α significance level of 5% was used, considering as statistically significant a p value less than .05.

Statistical analyzes were performed with R 2.15.2 software (R Development Core Team, 2014).

## RESULTS

### Descriptive analysis

The selected sample in this study consisted of 101 patients, 35 women (34.7%) and 66 men (65.3%). The average age of patients was 63.1 years, ranging from 27-83 years. Ninety-eight (97.0%) were Child A and the mean MELD score was 8.6, ranging from 6 to 26. 

Eleven patients had HBV (10,9%), 34 HVC (33.7%), 13 alcoholic liver disease (12.9%), eight NASH (7.9%), 14 had mixed etiology (13.9%) and the remaining 21 patients had other etiologies (20.8%).

It is worth mentioning that 77 patients had confirmed cirrhosis on pathology (76.2%).

Fifty-seven patients met the Milan criteria (58.2%). Patient's data are shown on Table 1.

**TABLE 1:** Disease characteristics among patients with HCC

		n	%
Etiology	HBV	11	10.9
	HCV	34	33.7
	alcohol	13	12.9
	NASH	8	7.9
	mixed	14	13.9
	others	21	20.8
	Total	101	100.0
Cirrhosis	yes	77	76.2
	no	24	23.8
	Total	101	100.0
Nodule (cm)	n	98	
	average	6.8	
	median	4.8	
	minimum-maximum	0.5-24.0	
	standard deviation	5.1	
Edmondson Steiner	I	1	1.0
	II	26	26.5
	III	65	66.3
	IV	6	6.1
	Total	98	100.0
Milan criteria	yes	57	58.2
	no	41	41.8
	Total	98	100.0
Capsule	yes	63	64.3
	no	35	35.7
	Total	98	100.0
Capsule invasion	yes	11	17.5
	no	52	82.5
	Total	63	100.0
Satellites	yes	13	13.4
	no	84	86.6
	Total	97	100.0
Vascular invasion	yes	52	53.6
	no	45	46.4
	Total	97	100.0
Free margin	yes	89	92.7
	no	7	7.3
	Total	96	100.0

Seventy-three patients underwent open (75.3%), while 24 laparoscopic surgery (24.7%); resection was anatomic in 69 patients (69.7%).

Postoperative complications were observed in 47.5% of the patients and ascites, ileus, infection, kidney and lung were the most common complications (Table 2). 

**TABLE 2:** Distribution of postoperative complications in patients undergoing resection of HCC

Complication	n	%
Yes	48	47.5
Clinical (ARF,BCP, PE, MI and others)	16	33.3
Intra-abdominal abscess	10	20.8
Ascites	8	16.7
Bile leak	8	16.7
Wound infection	7	14.6
Hemorrhage	6	12.5
Reoperation	5	10.4
Ileus	5	10.4

ARF=acute renal failure; BCP=bronchopneumonia; PE=pulmonary embolism; MI=myocardial infarction

### Survival analysis

Six patients died between the immediate postoperative period and up to a month after surgery and were excluded from the survival analysis. 

From 95 patients evaluated, there were 44 deaths (46.3%) and 51 patients (53.7%) were alive at the end of follow-up. Forty-six patients (48.4%) had disease recurrence.

**FIGURE 1:**
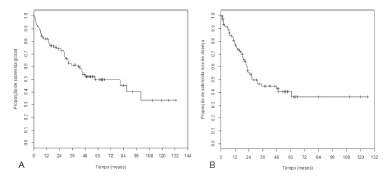
Patients undergoing resection for HCC: A) overall survival curve; B) disease-free

The overall survival curve (Figure 1A) shows that, within five years follow-up, 49.9% of patients remained alive. The average overall survival of patients who died was 24.6 months, ranging from 1 to 100 months.

For disease-free survival, at five years, 40.7% of patients showed no recurrence (Figure 1B). The average duration of disease-free survival of patients who relapsed was 17.2 months, ranging from 1.1 to 60.6 months.

**FIGURE 2:**
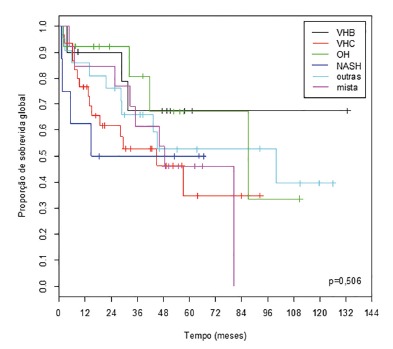
Overall survival curves of patients who underwent resection of HCC, according to the etiology of liver disease

At five years of follow-up, patient overall survival, according to the etiology of liver disease, in ascending order, was: HCV (34.8%), mixed (46.2%), NASH (50.0%), other (52.8%), alcohol (67.3%) and HBV (67.5%) (Figure 2).

**FIGURE 3:**
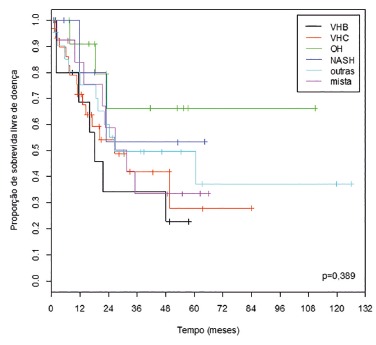
Disease-free survival curves of patients who underwent resection of HCC, according to the etiology of liver disease

At five years of follow-up, disease-free survival, according to the etiology of liver disease, in ascending order, was: HBV (22.9%), HCV (27.9%), mixed (33.6%), other (37,2%), NASH (53.3%) and OH (66.3%) (Figure 3). 

**FIGURE 4:**
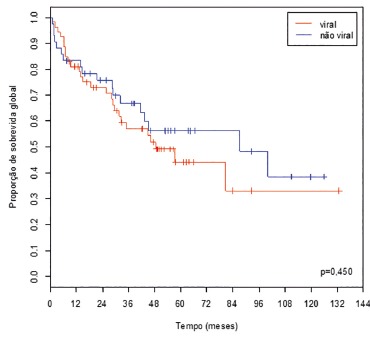
Overall survival curves of patients who underwent resection of HCC, according to the etiology (viral / non-viral) of liver disease

**FIGURE 5:**
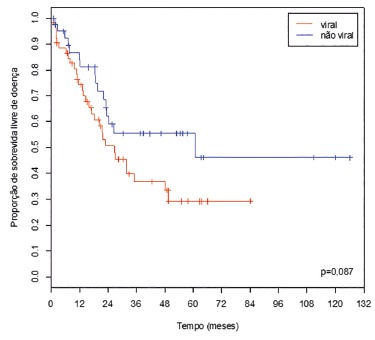
Disease-free survival curves of patients who underwent resection of HCC, according to the etiology (viral / non-viral) of liver disease

When it was proceeded to the same analysis, but dividing etiologies in viral and non-viral, the overall survival rates, at five years, were 44.3% and 56.3%, respectively (Figure 4). Disease-free survival rates, at five years, were 29.3% and 55.5%, respectively (Figure 5).

## DISCUSSION

Resection appears today as the main approach for treatment of HCC, especially in patients with good liver function. This mainly occurs because of the restrictive criteria and the inadequate supply of grafts for transplantation in the world^1^ ^,^ ^11^ ^,^ ^13^ ^,^ ^16^ ^,^ ^19^.

Fan et al. ^7^ showed that only about 2% of HCC patients have the possibility to be transplanted, while liver resection may be performed in about 25% of these patients.

The five-year overall survival of HCC resection is similar to liver transplantation, when considering intention to treat, being around 60-70%^19^. In Child A patients with a single nodule within Milan Criteria, the five-year overall survival after resection can be considered better than liver transplantation, especially if is made an analysis by intention to treat (considering also patients who died in the waiting list)^13^ ^,^ ^15^ ^,^ ^19^.

This study found an overall and disease-free survival at five years of 49.9% and 40.7%, respectively. One possible explanation for the low overall survival rate in this series is the fact that the sample was composed of more than 40% of patients out of the Milan criteria.

Tumor biology appears more and more as the protagonist, and makes us wonder why some tumors of the same histological type are more aggressive than others.

Some studies have shown a difference in the prognosis when considering the etiology of HCC patients who underwent liver resection^3^ ^,^ ^12^ ^,^ ^20^, while others have failed to reach the same conclusion^8^.

Chirica et al.^3^ have demonstrated in a sample of 75 patients, who underwent resection of HCC, poorer outcomes for patients with viral etiology, especially HCV. 

Zhou et al.^20^ performed a meta-analysis of 20 studies correlating etiology and prognosis and also concluded that the viral etiology carries a worse prognosis for resected patients^13^. There was a tendency toward greater overall and disease-free survival among patients with non-viral etiology, with no difference between those carriers of HBV and HCV.

Fong et al.^8^ found no difference in prognosis between viral and non-viral etiology patients in a 1999 study, but they attributed that to the small sample size and to the presence of only a few cases of some other etiologies. 

This study found no statistically significant difference between groups of different etiologies of HCC with respect to the prognosis. 

Regarding the overall survival at five years, was noted that patients whose etiologies were alcohol and HBV had the best results with 67.3% and 67.5%, respectively, while HCV patients had the worst results, with only 34.8% survival at five years.

When is looked at disease-free survival curves at five years, is observed that patients whose etiologies were alcohol and NASH showed the best results, with 66.3% and 53.3%, respectively, while viral etiologies (HCV and HBV) had the worst, 27.9% and 22.9%, respectively. 

These data are compatible with the literature, always showing a worse prognosis for viral etiologies and a better prognosis for alcoholic etiology. 

This is reinforced when is looked at the survival curves divided by viral status (Figures 4 and 5). Although there was no statistically significant difference, can be observed a trend to a better prognosis for non-viral patients, especially in regard to disease-free survival.

Another interesting finding in this analysis was the situation of HBV patients, who had one of the best overall survival results, along with alcoholic liver disease patients, but were among the worst in the evaluation of disease-free survival, together with the HCV patients.

This could be explained mainly by the different mechanism involved in hepatocarcinogenesis of HBV, which has the potential to integrate into the DNA of liver cells, causing changes that can lead to tumor development, even without the presence of cirrhosis. The other etiologies appear to act by direct aggression to the hepatocyte, leading initially to the development of cirrhosis and then HCC^20^. 

This could explain the possibility of development of HCC in non-cirrhotic patients infected with HBV. These patients, in theory, could have better overall survival compared to other etiologies because they have preserved liver function and are part of screening programs, which can facilitate detection of the disease at earlier stages, providing better overall survival results, but also higher rates of recurrence.

A limitation of this study was the relatively small size of this sample, which, despite representing a large sample from a surgical point of view, did not have the power to demonstrate significant differences in this study.

Thus, this study can serve as a basis for further research in order to confirm the findings and to better assess the results pointed out here, continuing the search for greater knowledge and improved care for patients with HCC.

## CONCLUSION

There was no statistically significant difference in overall and disease-free survival at five years among groups of patients with different etiologies of HCC who underwent liver resection with curative intent.
